# Odontoma Recurrence. The Importance of Radiographic Controls: Case Report with a 7-Year Follow-Up

**DOI:** 10.3390/medicina60081248

**Published:** 2024-07-31

**Authors:** Josefa Alarcón Apablaza, Gonzalo Muñoz, Carlos Arriagada, Cristina Bucchi, Telma S. Masuko, Ramón Fuentes

**Affiliations:** 1 Doctoral Program in Morphological Sciences, Faculty of Medicine, Universidad de La Frontera, Temuco 4780000, Chile; josefa.alarcon@ufrontera.cl (J.A.A.);; 2 Undergraduate Research Group in Dentistry (GIPO), Faculty of Health Sciences, Universidad Autónoma de Chile, Temuco 4780000, Chile; 3 Master Program in Dental Sciences, Dental School, Universidad de La Frontera, Temuco 4780000, Chile; 4 Oral Biology Research Centre (CIBO-UFRO), Dental School—Facultad de Odontología, Universidad de La Frontera, Temuco 4780000, Chile; cristina.bucchi@ufrontera.cl; 5 Department of Integral Adults Dentistry, Dental School, Universidad de La Frontera, Temuco 4780000, Chile; 6 Department of Biomorphology, Institute of Health Sciences, Bahia Federal University (ICS-UFBA), Salvador 402331-300, Brazil; tsmasuko@uol.com.br; 7 Research Center in Dental Sciences (CICO-UFRO), Dental School—Facultad de Odontología, Universidad de La Frontera, Temuco 4780000, Chile

**Keywords:** diagnostics, odontoma, recurrence, recidivism, complications, tomography, imaging

## Abstract

Odontomas are benign tumors characterized by slow and limited growth with a rare recurrence. Odontomas are generally detected by radiographic findings in the radiopaque stage, where calcification of the tissues is observed. This article seeks to report the recurrence of a radiologically diagnosed odontoma to show the importance of radiographic controls after enucleation as a diagnostic and follow-up method. Case report: A female patient, 9 years old, attended dental care in 2020 due to malpositioned teeth. In the intraoral clinical examination, she presented stage II mixed dentition with crowding. A radiographic exam showed no associated lesions. The patient reported a history of odontoma removal and a supernumerary tooth in sextant II in 2016. Subsequently, she was referred to orthodontics, where permanent dentition with moderate anterior crowding in the maxilla and mandible was observed. The radiographic examination showed a radiopaque area compatible with odontoma, palatal to teeth 12 and 13. Conclusions: Although recurrence is rare, complete removal in the case of an odontoma is critical. This study demonstrates the importance of performing radiographic controls 5 years after enucleation of an odontoma, considering the stages of evolution.

## 1. Introduction

Odontogenic tumors constitute about 3% of oral lesions [1]. Among these, odontomas are the most frequent, accounting for 22% [2,3]. The term “odontoma” was introduced by Pierre Paul Broca in 1867, referring to any tumor formed by transient or complete overgrowth of dental tissues [4]. Odontomas are classified as odontogenic tumors; however, due to their slow and limited growth, they are considered hamartomas [5,6,7,8]. They originate from an alteration of differentiated epithelial and mesenchymal odontogenic cells that can form enamel, dentin, and cementum [5,6,9]. Trauma during primary dentition, genetic factors, cell rests of Serres, and genetic mutations are accepted as possible etiological factors. Odontomas have also been associated with pathological conditions, inflammatory processes, and/or infections [4]. 

The World Health Organization classifies odontomas according to the degree of differentiation as (1) compound odontomas and (2) complex odontomas. The former are approximately twice as common as complex odontomas [5]. The compound odontoma is usually located in the anterior maxilla and appears radiographically as small, multiple, irregular, radiopaque, tooth-like denticles in the center of a radiolucent lesion. They consist of enamel, dentin, cementum, and pulp tissue [4,6]. The complex odontoma is usually found in the posterior mandibular region as an amorphous calcification of dental tissues arranged in an irregular pattern, with no resemblance to a tooth structure, surrounded by a thin radiolucent demarcation line [4,6,8]. 

The odontoma goes through stages similar to developing teeth. In the early stages, odontomas may appear radiolucent. This is due to less dense tissue, such as pulp, and areas of non-mineralized tissue. This is followed by an intermediate stage, characterized by partial calcification of the odontogenic tissues, producing a radiolucent–radiopaque picture. Finally, the most radiopaque stage is reached, in which the calcification of the dental tissues is complete. Odontoma formation begins during infancy, coinciding with the development of the natural dentition [5,10,11]. 

Most odontomas are asymptomatic, although there are occasionally signs and symptoms related to their presence [4]. The most frequent reason for a consultation of a patient with an odontoma is delayed tooth eruption. However, odontomas are frequently detected during routine radiographic examinations [12]. 

Recurrence of odontomas is very rare [5,13,14,15,16]. It is generally not recommended to perform surgical procedures for enucleation before the age of 5, due to several factors. One of the main reasons is the risk of enucleating permanent dental follicles, which could affect the normal development of permanent teeth that have not yet erupted [17,18]. Furthermore, there is an increased risk of recurrence from these tissues following surgery [13]. This is because odontomas can be closely associated with surrounding tissues, and surgery on a developing bone structure can be more complicated, increasing the likelihood that remnants of the odontoma will remain. It is very important to remove the surrounding capsule, as any remnants can increase the risk of lesion recurrence [5,13]. However, complete removal of the lesion is difficult in the early stages of development, due to odontomas being characterized by the presence of non-calcified cellular portions [13]. In light of the above, this article aims to document the recurrence of a radiologically diagnosed odontoma, to highlight the importance of radiographic controls after enucleation as a diagnostic and follow-up method.

## 2. Case Report

The case presentation is documented chronologically. Authorization to publish the case was obtained, with informed consent from the mother and the patient. 

**Figure 1:**
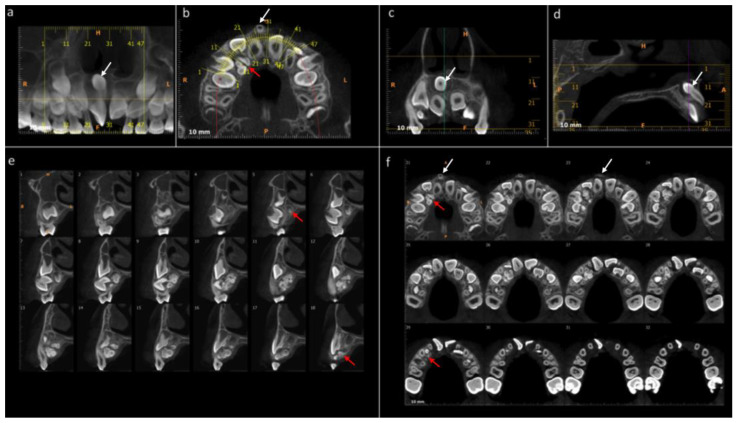
Cone-beam computed tomography (CBCT) of the maxilla. 2016, 6 years old, prior to first surgery. (**a**) The panoramic reconstruction shows the presence of a mesiodens supernumerary tooth between 11 and 21. (**b**) Axial section. Location of mesiodens vestibular to teeth 11 and 21. Presence of multiple denticles compatible with odontoma, in intraosseous evolution, distal to tooth 11, and palatal to teeth 12 and 13. (**c**) Coronal section. Presence of mesiodens in relation to teeth 11 and 21, surrounded by a radiolucent demarcation area. (**d**) Sagittal section. Presence of supernumerary, crown in apical position and root in coronal area. Presence of pericoronary sac. (**e**) Sagittal section. Presence of multiple radiopaque masses with irregular margins compatible with odontoma (Sections 5–18) in intraosseous evolution palatal to tooth 13. (**f**) Axial section. Presence of mesiodens (Sections 21–23) in vestibular relation to teeth 11 and 21. Presence of radiopaque masses compatible with odontoma (Sections 21–29) distal to tooth 11 and palatal to teeth 12 and 13. White arrow = mesiodens. Red arrow = odontoma.

In 2020, a 9-year-old female patient attended dental care. The reason for consultation was tooth malpositioning. There was no indication of systemic disease or medication in the medical history. The patient reported the surgical removal of an odontoma and supernumerary in the anterosuperior sector in 2016, diagnosed radiographically (Figure 1). 

The maxillary cone-beam computed tomography (CBCT) exam performed in 2016, prior to the first surgery, detected a mesiodens in an intraosseous formation, in vestibular relation to teeth 11 and 21, distoangular, with the crown in the apical position and the root in the coronal zone. In addition, multiple radiopaque masses with irregular margins compatible with an odontoma were observed in the palatal area about teeth 11, 12, and 13 (Figure 1). The odontoma and mesiodens were surgically removed in the same year as their diagnosis. 

**Figure 2:**
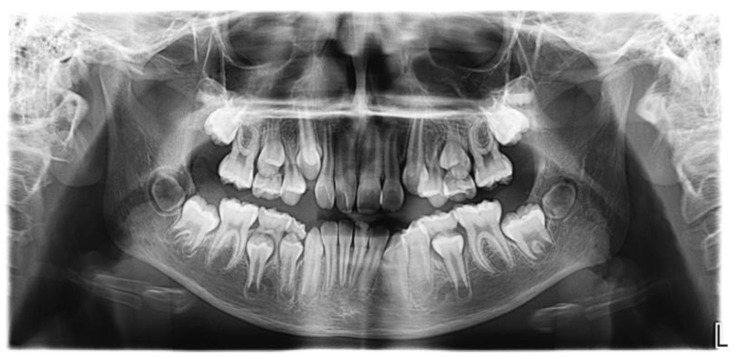
Panoramic X-ray. 2020, four years after the first surgery. Maxillary sinuses of ample development, shape, contour, and characteristic transparency. Stage II mixed dentition. 13, 43, and 33 lack radiographic space for a correct position in the dental arch. Third molars in intraosseous evolution.

In the intraoral clinical examination performed in 2020, four years after the first surgery to remove the odontoma and mesiodens, the patient presented stage II mixed dentition with a normal eruption time and chronology. Rotation was noted in teeth 11 and 12. The panoramic X-ray (Figure 2) showed a lack of radiographic space for correct positioning of teeth 13, 33, and 43 in the dental arch, rotation of teeth 11 and 12, total root resorption of teeth 55, 65, 75, and 84, almost total root resorption in tooth 85, intraosseous evolution of teeth 17, 27, 35, 37, 45, and 47, and extraosseous evolution of teeth 13, 15, 23, 25, 33, 34, 43, and 44.

Due to health contingencies around the SARS-CoV-2 virus, the patient postponed orthodontic treatment until 2021. 

**Figure 3:**
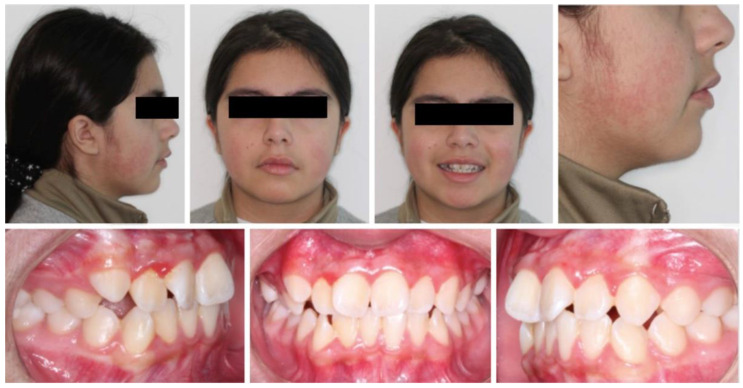
Extraoral and intraoral photographs for diagnosis and corrective orthodontic treatment planning.

The patient attended dental care in February 2021 for corrective orthodontic treatment. Extraoral examination showed vertical and horizontal symmetry within normal parameters, lips together in the resting position, normal nasolabial and mentolabial angles, and slight lip protrusion. Intraoral examination showed permanent dentition with moderate crowding in the anterior region of the maxilla and mandible (Figure 3). 

**Figure 4:**
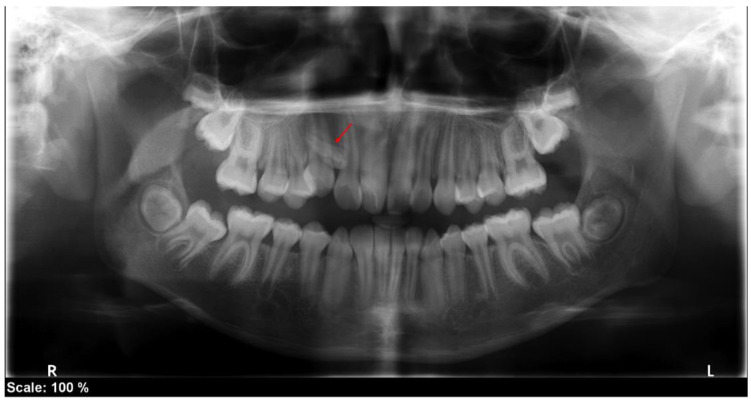
Panoramic X-ray. 2021, five years after the first surgery. Presence of a radiopaque element with a denticle shape in relation to teeth 12 and 13. Red arrow = odontoma.

**Figure 5:**
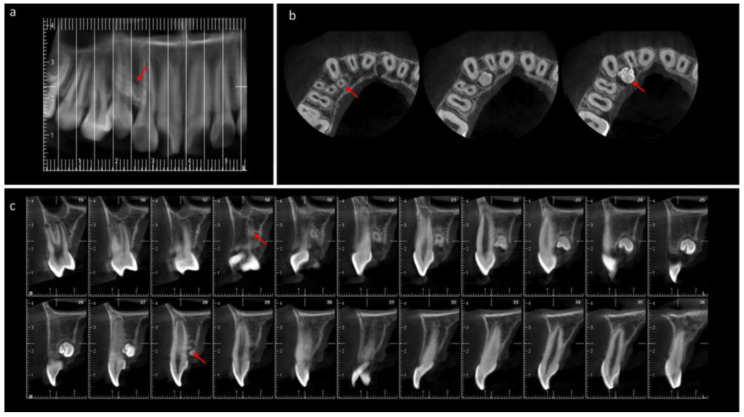
Cone-beam computed tomography (CBCT) of the maxilla. 2021, five years after the first surgery. (**a**) Panorex section. Presence of a radiopaque area, compatible with odontoma, overprojected to teeth 12 and 13, mesioangular. (**b**) Axial section. Multilobulated odontoma, located palatal to teeth 12 and 13, surrounded by a radiolucent demarcation line. Discrete external resorption in the cervical third of the palatal root of tooth 13. (**c**) Cross-section. Presence of odontoma (Sections 18–28) in intraosseous evolution, microdontic, mesioangular, coronal structural alteration. Palatally displaced tooth location with thinning and perforation of palatal bone cortex. The crown is located palatally and in contact with the cervical third palatal root of tooth 12 and the cervical and middle third palatal root of tooth 13. Root location is palatally displaced. Root middle third in contact with palatal root middle third of tooth 13. Pericoronary sac and periodontal ligament space of preserved thickness (scale 100%). Red arrow = odontoma.

A radiopaque element with a denticle form of intraosseous evolution was observed in the radiographic update of the diagnostic studies carried out to begin the orthodontic treatment examination, performed five years after the first surgery to remove the odontoma and mesiodens (Figure 4). CBCT was requested to confirm the finding and plan the surgical removal. A radiopaque area compatible with an odontoma was observed in relation to teeth 12 and 13 in a marked mesioangular position, microdontic, located palatal to the roots of the neighboring teeth, and in intraosseous evolution (Figure 5). The odontoma was surgically removed. 

**Figure 6:**
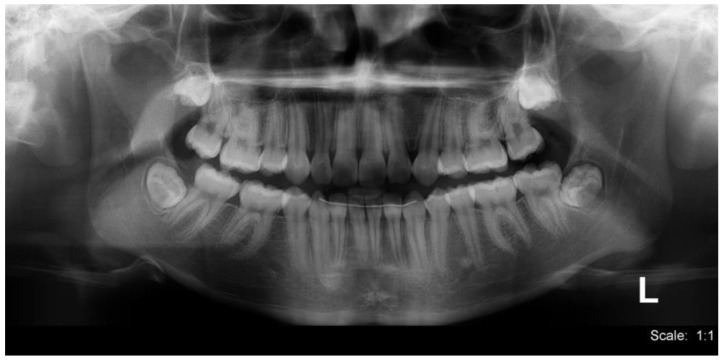
Panoramic X-ray. 2023, 24 months after the second surgical removal. Permanent dentition. Third molars in intraosseous evolution. Anterior mandibular splinting.

Twenty-four months after surgical removal of the odontoma recurrence, a radiographic control was performed, where no apparent recurrence was observed (Figure 6). 

## 3. Literature Review

### 3.1. Systematic Literature Search

A review was performed on odontoma recurrence. Our review was performed according to the Preferred Reporting Items for Systematic Reviews and Meta-Analyses extension for Scoping Reviews (PRISMA-ScR) guidelines [19]. 

An electronic search was conducted in three digital databases (PubMed, SCOPUS, and Web of Science). The search was performed between January and March 2024. The bibliographies of potentially eligible clinical trials, case reports, case studies, and systematic reviews were also screened for additional studies that were possibly fit for inclusion. The following search equation was used in PubMed:

(“Odontoma” [MeSH Terms] OR (“Odontoma” [MeSH Terms] OR “Odontoma” [All Fields] OR “odontomas” [All Fields]) OR “odontoma compound” [All Fields] OR “odontomas compound” [All Fields] OR (“tooth abnormalities” [MeSH Terms] OR (“tooth” [All Fields] AND “abnormalities”[All Fields]) OR “tooth abnormalities” [All Fields] OR “odontome” [All Fields] OR “odontomes” [All Fields]) OR “odontome*” [All Fields]) AND (“Recurrence” [MeSH Terms] OR “neoplasm recurrence, local” [MeSH Terms] OR (“reappear” [All Fields] OR “reappearance” [All Fields] OR “reappearances” [All Fields] OR “reappeared” [All Fields] OR “reappearing” [All Fields] OR “reappears” [All Fields]) OR (“recurrance” [All Fields] OR “Recurrence” [MeSH Terms] OR “Recurrence” [All Fields] OR “recurrences” [All Fields] OR “recurrencies” [All Fields] OR “recurrency” [All Fields] OR “recurrent” [All Fields] OR “recurrently” [All Fields] OR “recurrents” [All Fields]) OR (“recrudesce” [All Fields] OR “recrudesced” [All Fields] OR “recrudescent” [All Fields] OR “recrudescing” [All Fields] OR “Recurrence” [MeSH Terms] OR “Recurrence”[All Fields] OR “recrudescence” [All Fields] OR “recrudescences” [All Fields]) OR (“recrudesce” [All Fields] OR “recrudesced” [All Fields] OR “recrudescent” [All Fields] OR “recrudescing” [All Fields] OR “Recurrence” [MeSH Terms] OR “Recurrence” [All Fields] OR “recrudescence” [All Fields] OR “recrudescences” [All Fields])).

### 3.2. Eligibility Criteria

Observational (case reports and case series) and experimental (randomized and controlled clinical trials) studies were included where they reported odontoma recurrence. The potentially eligible articles were screened based on the inclusion criteria: studies in English or Spanish and a full text with no publication date limit. A summary of the inclusion and exclusion criteria considered in this review is given in Table 1.

### 3.3. Article Selection and Data Extraction

Two independent reviewers analyzed articles obtained in the systematic search process by reviewing the titles and abstracts. The articles that met the eligibility criteria were then analyzed in their full text to confirm their relevance. 

**Figure 7:**
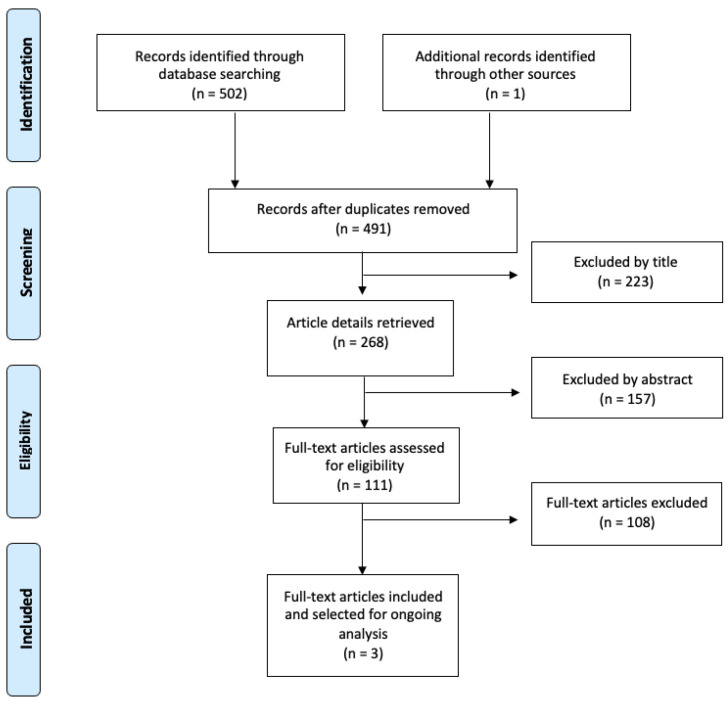
Flow chart for study selection.

The article search and selection process is summarized in Figure 7. The total number of articles found in the databases was 502, and 1 was identified from the manual search, of which 12 were duplicates. After the initial reading by title, 223 were discarded, of which 112 were studies that reported only the characteristics, diagnosis, treatment, or surgical management of odontoma, 64 articles studied other tumors, 37 were not related to the subject under study, and 10 were systematic reviews. Subsequently, 157 studies were discarded due to the abstract, of which 76 were studies that reported only the characteristics, diagnosis, treatment, or surgical management of odontoma, 68 were not related to the subject under study, and 13 were systematic reviews. After reading the full-text articles (111 articles), 108 were excluded, of which 68 were studies that reported only the characteristics and surgical management of odontoma, 21 reported other tumors, and 19 were systematic reviews. Finally, in this review, 3 articles corresponding to observational and experimental studies that reported odontoma recurrence were included [3,7,20].

The data were extracted from the reports of the selected cases, and information considered relevant for the analysis is shown in Table 2.

## 4. Discussion

This case report describes the recurrence of a radiologically diagnosed odontoma to highlight the importance of radiographic controls after enucleation as a method of diagnosis and follow-up. Nonetheless, although the radiological approach provides valuable diagnostic information, we recognize that histopathological analysis remains the gold standard to confirm the diagnosis and ensure appropriate treatment. Omitting histopathological analysis could have significant implications, including the possibility of residual diagnostic uncertainty and the risk of not detecting other potential pathologies. To mitigate these consequences, we consider it crucial to extend the patient’s follow-up time. This measure will allow continued surveillance for any possible recurrence or complications that may arise.

Conventional X-rays, such as periapical and panoramic, are often considered the gold standard in detecting and evaluating odontomas. However, CBCT is a valuable tool for ascertaining the precise location of the odontoma with respect to adjacent teeth [13,21]. Following diagnosis and localization, the treatment of choice for odontomas is surgical removal. However, special care must be taken to remove it completely to avoid recurrence [4]. Although the odontoma is a common odontogenic tumor [1], its recurrence is rare [2,3]. Only three studies were found reporting odontoma recurrence [3,7,20]. 

The main possibilities for recurrence are an odontoma and secondary ossifying fibroma (OF) [20]. Radiographic differentiation between an odontoma and an ossifying fibroma is crucial for the diagnosis of these lesions. Odontomas are typically radiopaque, appearing as white areas on radiographs due to their enamel and dentin content. They may show a “tooth” appearance within a mass, especially in compound odontomas, whereas complex odontomas are disorganized masses of dental tissues [4,6]. In addition, odontomas often have a well-defined halo and may be surrounded by a radiolucent capsule, indicating the presence of soft fibrous tissue around the tumor. Radiographically, a compound odontoma appears as a mixed image (radiopaque and radiolucent), which adopts a configuration of multiple clearly distinguished denticles surrounded by a radiolucent halo; and complex odontomas appear as one or multiple radiopaque masses surrounded by a radiolucent halo [4,6,22,23]. In contrast, the density of the FO mass is even; it can vary from radiolucency to a mixture of radiolucency and radiodensity, due to the presence of fibrous tissue and calcifications [24,25,26]. The boundary between the lesion and the surrounding bone is not clearly discernible [27]. Unlike odontomas, FO has a lytic appearance, compromising the bone and causing bone expansion. It is also common to observe root resorption and displacement of involved teeth. FO can be unilocular or multilocular [24,25,26]. These distinctive radiographic features allowed us to differentiate that the recurrence in this case was an odontoma, although histopathological analysis remains the gold standard to confirm the diagnosis and ensure adequate treatment

Recurrence occurs when resection is performed at the noncalcified stage of the lesion or if the resection is incomplete [3,28]. In the present report, radiographic images performed in 2016 indicated that the odontoma was in an advanced developmental stage of mineralization, as a radiopaque stage was observed, with multiple denticles. Therefore, the recurrence must have been due to a residual mass in the first enucleation [3,28] or the presence of the capsule surrounding it [5,13]. In the radiological analysis performed 4 years after the enucleation, the odontoma was at an early stage of development and was not calcified. Radiolucency makes it difficult to visualize a lesion [7], which can cause the undiagnosed recurrence of an odontoma. Here, at one year, the radiolucent lesion had progressed to a radiopaque mineralized stage, which made the recurrence diagnosis possible 5 years after enucleation of the odontoma, similar to the follow-up time of recurrences reported in the literature [3,7,20].

The follow-up time after enucleation of an odontoma is an important factor to consider. Several studies report no recurrence; however, they do not exceed 3 years of follow-up after surgical removal [2,17,29,30,31,32,33,34,35,36,37,38]. It is important to consider the stages of evolution of odontoma since, in the early stages of development, it is very difficult to diagnose [39]. In the cases reported in the literature, most odontomas appeared as well-calcified masses on the X-rays [4]. However, some cases were not diagnosed in the first radiographic examination [7]. In a retrospective study of X-rays taken at different times, Tomiwasa et al., 2005, revealed that calcification was still immature in the early stages of odontoma development with a radiolucent image being observed, making it very difficult to diagnose. This becomes very important since it is clear that, from the earliest stages, an odontoma may disturb tooth eruption and lead to complications [7]. 

Various complications have been reported for odontomas, such as displacement of teeth surrounding the odontoma, delayed eruption of permanent teeth [3], diastemas, displacement, rotation, crowding, rhyzolisis, periodontal lesions, or even pulp necrosis of adjacent teeth [40,41]. Therefore, although recurrence of an odontoma is very rare, it can lead to complications if not diagnosed promptly [7,41]. In the present case, due to the radiographic control, a timely diagnosis of recurrence could be made, and complications were avoided. 

Early detection of an odontoma is more likely to be a radiological finding, so the need for routine radiographic analysis should be emphasized. Odontomas have been reported to have limited growth potential and a slow-growing clinical course [41]. Hence, based on the evolution time of the reported case and what is reported in the literature, a time of 5 years after enucleation of the odontoma is suggested.

This study has certain limitations. It is predicated on the presentation of a single case report, and more cases should be evaluated to draw more significant conclusions regarding the control period after odontoma removal. However, the rarity of odontoma recurrence makes obtaining larger samples difficult. Therefore, this case report provides a basis for establishing an appropriate follow-up period. It is also important to note that the diagnosis of odontomas in this study was mainly based on radiographic interpretation, which could limit the accuracy of the diagnosis compared to histological analysis. Although X-rays are a useful tool in the initial detection of odontomas, it is recognized that definitive diagnosis should ideally be confirmed by histological analysis for a more complete and accurate characterization of the lesion.

## 5. Conclusions

Here, the complex analysis and follow-up made it possible to confirm the recurrence of the odontoma by radiographic diagnosis. Surgical treatment obtained a satisfactory result in the medium and long term, confirmed by the periodic radiographic reevaluation protocol, which was established using a correct initial diagnosis and following the suggestions proposed in the literature. 

This study guides dentists to perform radiographic controls 5 years after enucleation of an odontoma, considering the stages of evolution. This will allow for an early diagnosis in patients who do not report clinical signs or symptoms, avoid complications, and enable treatment planning according to the location and associated anatomical structures of each case.

## Figures and Tables

**Table 1:** Inclusion and exclusion criteria of the scoping review.

Inclusion Criteria	Exclusion Criteria
Observational and experimental studies	Systematic literature reviews or letters to the editor
Studies that report odontoma recurrence	Studies that report only the characteristics, diagnosis, or treatment of odontoma
English or Spanish language, and full text with no publication date limit	Studies reporting another tumor

**Table 2:** Case reports of odontoma recurrence reported in the literature.

Study	Type of Study	Tooth Involved	Age of First Enucleation	Age of Recurrence	Diagnosis of Recurrence	Complications
Boffano, P., 2022 [3]	Multicenter study with 4 recurrences in 127 patients	Not reported	22 years on average	Not reported	Odontoma	Not reported
Tomizawa, M., 2005 [7]	Case report in 1 subject	Left maxillary primary central incisor	1 year and 8 months	6 years and 5 months	Odontoma	Delayed eruption
Matsuo, K., 2013 [20]	Case report	Second deciduous molar	3 years	7 years	Ossifying fibroma	Delayed eruption

## Data Availability

Data is contained within the article.
